# Complete heart block and moderate stenosis aortic post radiation in a young woman with breast cancer

**DOI:** 10.1016/j.amsu.2022.103505

**Published:** 2022-05-06

**Authors:** Ichraq Bourouis, Lamyae Zinoune, Oumayma Hattab, Saîda Amaqdouf, Noha El Ouafi, Zakaria Bazid

**Affiliations:** a Department of Cardiology, Mohammed VI University Hospital, Faculty of Medicine and Pharmacy of Oujda, Mohammed First University, Oujda, Morocco; b Epidemiological Laboratory of Clinical Research and Public Health, Faculty of Medicine and Pharmacy of Oujda, Mohammed First University, Oujda, Morocco

**Keywords:** Breast cancer, Complete heart block, Valve disease, Radiation therapy, Case report

## Abstract

**Introduction:**

and importance: Although radiotherapy is a well-known cancer treatment and an important part of the therapeutic strategy for achieving long-term remission or disease control, the radiation-induced heart disease rates are high and may occur years later. This article aims to raise clinician awareness of cardiac side effects that can occur years after radiation therapy. In order to develop effective prevention strategies and improve clinical outcomes.

**Case presentation:**

Here, we present a rare case of a young female, on remission from breast carcinoma, who received mediastinal radiotherapy 13 years earlier, admitted in our department for recurrent syncope of sudden on-set. The etiology of heart block was attributed to the distant effect of radiation-induced cardiac toxicity based on her past medical history.

**Clinical discussion:**

Radiation promotes fibrosis in all cardiac components, raising the risk of coronary artery disease, cardiomyopathy, valvulopathy, arrhythmias, and pericardial illness. In this population, physicians should aggressively address additional cardiovascular risk factors, and recommendations recommend obtaining routine imaging once symptomatology is established.

**Conclusion:**

Serious cardiovascular complications may develop several years after radiation treatment, Screening, early recognition, prevention and the use of certain drugs can be quite helpful in reducing radiation-induced heart damage.

## 1 Introduction

Radiotherapy is an important cancer treatment modality that has resulted in improved survival in patients with a variety of cancers [1].

Radiation can damage the heart through fibrosis of all components of the heart [2], and the major risk factor is the total dose of mediastinal radiation received. However, complications are often seen with any dose [3].

Radiotherapy can induce early complications such as myocarditis and pericarditis. Late complications include pericardial disease, cardiomyopathy, valvopathy, arrhythmias and coronary artery disease (CAD), in addition to medium and large vessel vasculopathy [4]. Conduction abnormalities, however, are uncommon and is limited to case reports and small series. Up to 4–5% of patients receiving thoracic radiation will develop conduction system pathology [5].

Our case report was written according to SCARE guidelines [6].

## 2 Case report

A 40 -year-old woman with a history of invasive ductal carcinoma of the left breast, underwent mastectomy and post-operative local radiotherapy 13 years ago (the total radiation dose was 46 Gy), came to our department for three episodes of syncope of sudden onset over the course of three weeks.Without history of heart disease, drug history, psychosocial history and family history including any relevant genetic information.

Each episode lasted for 4–10 seconds, with no associated chest pain.

On physical examination, the patient's vitals revealed a bradycardia 33-beat-per-minute pulse, a blood pressure of 96/55 mm Hg, oxygen saturation 96% on room air. Cardiovascular and other systemic examination was unremarkable.

**Fig. 1:**
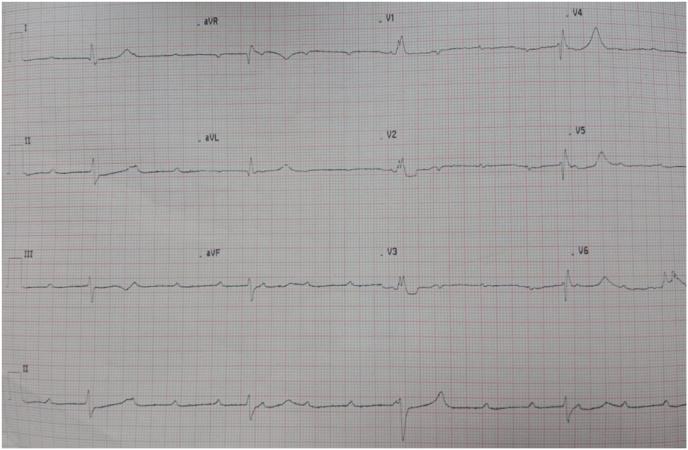
ECG showed a complete high grade atrioventricular block.

An electrocardiogram showed complete high-grade atrioventricular block with a heart rate of 25 bpm, no ST segment or T waves abnormalities were noted (Fig. 1).

**Fig. 2:**
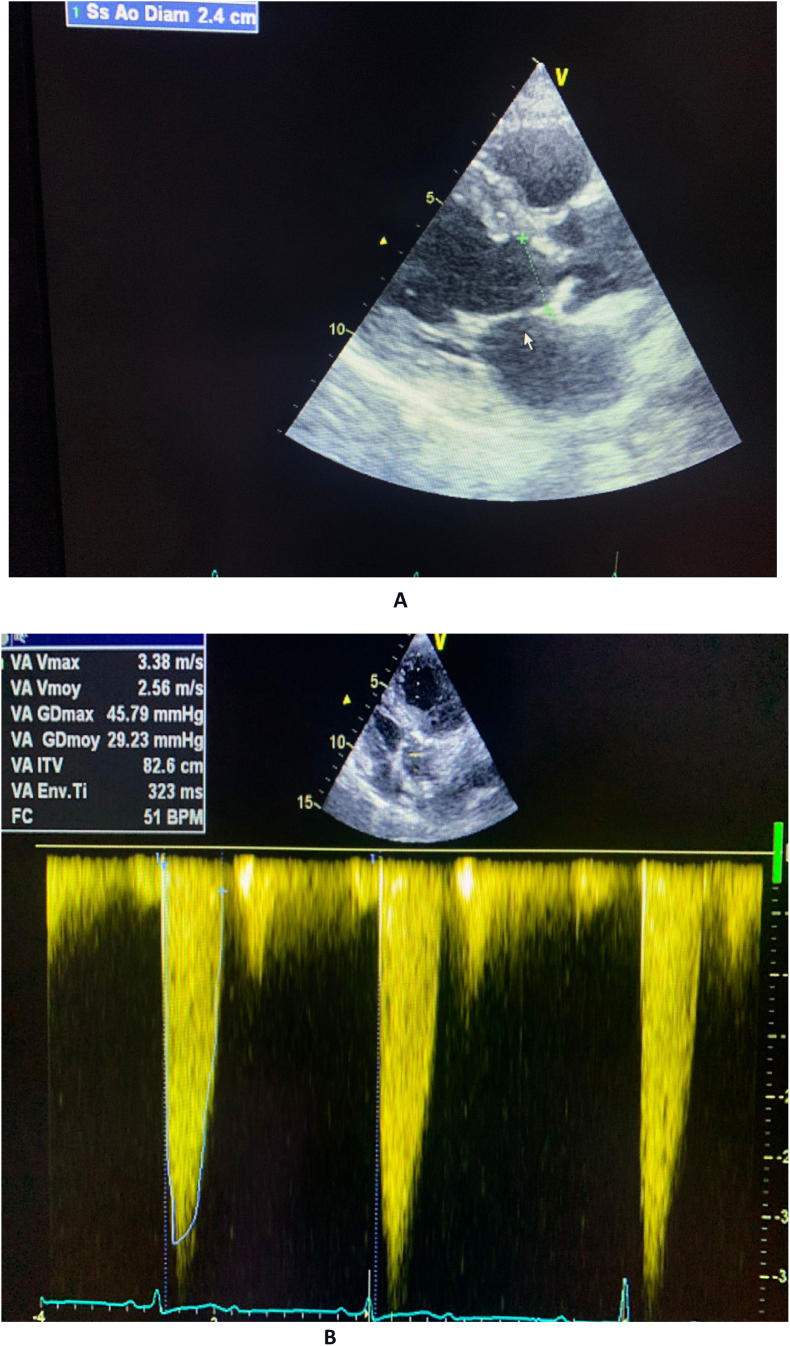
**A, B: Baseline echocardiogram:** Preserved LV function with moderate aortic stenosis.

Transthoracic echocardiography showed normal systolic and diastolic function, left ventricular (LV) ejection fraction of 60%, severe calcification of the aortic valve, with resultant moderate aortic stenosis (aortic valve area = 2,5 cm2. Vmax 3.38 m/s, medium gradient:30 mmhg) and normal right ventricle systolic function. (Fig. 2).

**Fig. 3:**
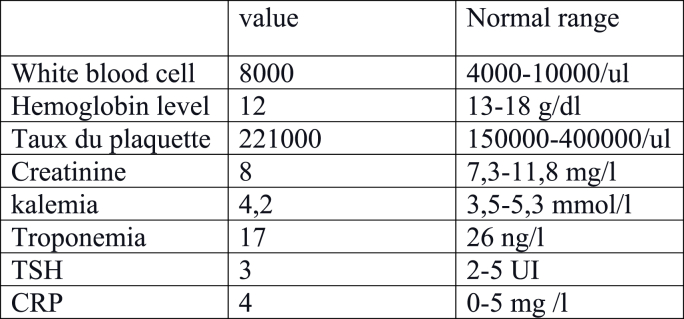
Blood investigations.

High sensitivity troponin and thyroid function were normal. Routine blood investigations including hemogram, coagulation parameters, renal function tests were normal too (Fig. 3).

The patient had dual-chamber pacemaker implantation successfully by an experienced professor of cardiology.

**Fig. 4:**
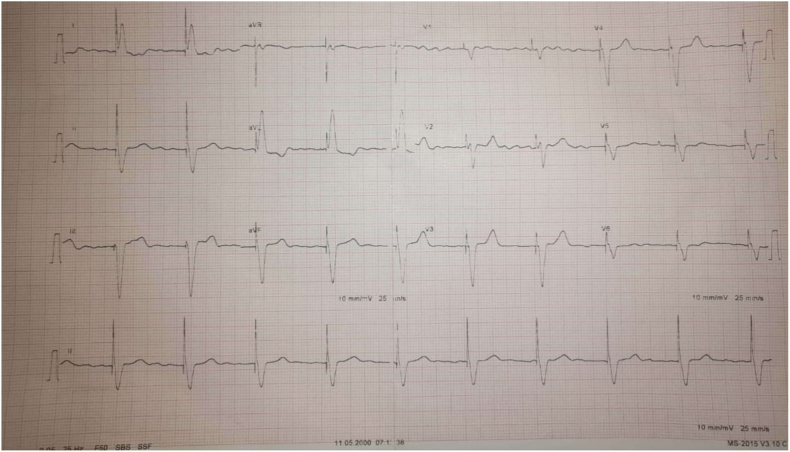
Post-procedural ECG showed a ventricular paced rhythm.

Post-procedural ECG showed a ventricular paced rhythm (Fig. 4).

## 3 Discussion

Breast cancer is the most common cancer in women, with approximately 40,000 women per year are expected to die from breast cancer [7].

Because a larger portion of the heart is included in the radiation field, complications are more common in patients with left-sided breast cancer than in those with right-sided breast cancer [8].

Patients who have undergone radiotherapy now have a 1.7 to 2-fold increased risk of cardiovascular death among cancer survivors [9].

Valvular conditions due to radiation range from mild asymptomatic valvular thickening to severe, hemodynamically significant valvular thickening, and may manifest as stenosis or insufficiency [10].

Valve cusps and leaflets undergo fibrotic changes and thickening as a result of radiation, with or without calcification. Regardless of the dose distribution, mediastinal radiation usually affects the left-sided valves [11].

The aortic valve is most commonly affected given its proximity with the radiation field, and AS is the most common valve pathology requiring correction [12].

Abnormalities of the entire conduction system, including varying degrees of atrioventricular block, sick sinus syndrome, prolonged QTc, supraventricular arrhythmias, and ventricular tachycardia, have been described in the context of radiotherapy [13,14]. Post-RT, right bundle branch block is more common than left bundle branch block, owing to the right ventricle's anterior location, which exposes it to more radiation.

Conduction abnormalities typically appear several years after radiation therapy. A 10- to 20-year delay after treatment completion has been reported [15].

In RIHD, few researchers have focused on the conduction system's mechanisms.

Radiation can damage the bundle either directly or indirectly through myocardial fibrosis and ischemia. Complete atrioventricular (AV) block is one of the most serious symptoms [2].

The exact treatment for radiotherapy-induced cardiac conduction abnormalities is determined by the nature and severity of the abnormalities. Complete atrioventricular dissociation (as in our case) necessitates the use of a permanent pacemaker. If the complete heart block is associated with left ventricular systolic dysfunction, cardiac resynchronization therapy (CRT-P) may be the better option. Because of the nature of cardiovascular manifestations that appear decades after initial radiotherapy, these patients should be followed for the rest of their lives [16].

Our patient was seen in the consultation of our university hospital one month after the discharge, he had no recurrence of the symtomatology.and ECG showed a ventricular paced rhythm.

Our patient was satisfied with the quality of our management.

For the time being, there is no effective treatment for radiation-induced cardiovascular disease RIHD, particularly in terms of preventing overexposure. Primary prevention is thought to be improvements in radiation regimens that reduce exposure to normal healthy tissue near tumor cells.

In order to reduce the incidence of this complication, a variety of techniques have been developed for minimizing radiation dose and volume received by the heart during radiotherapy have been used in clinical practice, such as deep inspiratory breath hold (DIBH), three-dimensional conformal radiotherapy, intensity modulated radiation therapy, and volumetric arc therapy [17,18].

It's may be impossible for cardiovascular tissues to be fully protected, secondary prophylaxis is essential. Cardiovascular risk factors such as hypertension, dyslipidemia, diabetes, obesity, inactivity, and smoking should be screened and aggressively treated in radiation patients. Some drugs, such as statins, ACE inhibitors, and antioxidants, have been shown in studies to help reduce radiation-induced cardiovascular disease RIHD [19].

In 2013, the American Society of Echocardiography proposed guidelines for the screening of radiation-induced cardiovascular disease, recommending that patients who have received more than 35 Gy of radiation be screened with a transthoracic echocardiogram, cardiac magnetic resonance imaging, or coronary computed tomography angiography, either 5 years after completion of therapy or after the age of 30–35 years old. Any new cardiac symptoms should be investigated [20].

On the other hand, additional study needs to be conducted to seek an optimal balance between protection of heart and assurance of entire breast tissue irradiation.

## 4 Conclusion

Tumor disease survival has improved as a result of therapeutic advances and early detection. Several years after the initial radiation treatment, serious cardiovascular complications may develop. The cardiac effects of radiation therapy are highlighted in this case. Our patient had multiple cardiac complications as a result of previous oncologic treatment for breast cancer, which manifested as valvular and conductive heart disease. With the rising risk of cancer as people get older and the prevalence of cardiac disease among the elderly, more research is needed to risk-stratify patients before they start radiotherapy. More research is needed to better understand the pathophysiology of cardiovascular disease after radiotherapy.

## Ethical approval

The ethical committee approval was not required give the article type (case report). but we notice that the written consent to publish the clinical data of the patients was given and is available to check by the handling editor if needed.

## Sources of funding

This research did not receive any specific grant from funding agencies in the public, commercial, or not-for-profit sectors.

## Author contribution

Ichraq Bourouis: Study concept, Data collection, Data analysis, Writing the paper, Lamiae Zinoune: Data analysis, El Ouafi Nouha: Conception, methodology, supervision, Bazid Zakaria: Conception, methodology, supervision.

## Trail registry number

This is a medical case report not an original research project. This registration is not required.

## Guarantor

Ichraq Bourouis.

## Consent

Written informed consent was obtained from the patient for publication of this case report and accompanying images. A copy of the written consent is available for review by the Editor-in-Chief of this journal on request.

## Provenance and peer review

Not commissioned, externally peer-reviewed.

## Declaration of competing interest

There are no conflicts.

## Declaration of competing interest

The authors state that they have no conflicts of interest for this report.
